# Association Between Chronic Renal Disease and the Risk of Glaucoma Development: A 12-year Nationwide Cohort Study

**DOI:** 10.1167/iovs.62.6.27

**Published:** 2021-05-27

**Authors:** Hyun-Kyung Cho, Jong Chul Han, Jin A. Choi, Jae Eun Chae, Rock Bum Kim

**Affiliations:** 1 Department of Ophthalmology, Gyeongsang National University Changwon Hospital, Gyeongsang National University, School of Medicine, Changwon, Republic of Korea; 2 lnstitute of Health Sciences, School of Medicine, Gyeongsang National University, Jinju, Republic of Korea; 3 Department of Ophthalmology, Samsung Medical Center, Sungkyunkwan University School of Medicine, Seoul, Republic of Korea; 4 Department of Ophthalmology, St. Vincent's Hospital, College of Medicine, Catholic University of Korea, Suwon, Republic of Korea; 5 STAT Department, LSK Global Pharma Services, Seoul, Republic of Korea; 6 Regional Cardiocerebrovascular Disease Center, Gyeongsang National University, Jinju, Republic of Korea

**Keywords:** chronic renal disease (CRD), glaucoma, longitudinal cohort study, nationwide cohort study

## Abstract

**Purpose:**

The purpose of this study was to present the results of our investigation into the risk of glaucoma development in patients with chronic renal disease (CRD).

**Methods:**

The present retrospective cohort study used the Korean National Health Insurance Service data, which consisted of 1,025,340 random subjects who were tracked from 2002 to 2013. Newly diagnosed glaucoma and CRD were included on the basis of the Korean Classification of Disease codes. The CRD group consisted of patients who received an initial CRD diagnosis between January 2003 and December 2007 as an index period (*n* = 3640). The control group (*n* = 17,971) was selected using 1:5 propensity-score matching using social and demographic factors, along with the year of enrollment. Each group subject was followed until 2013. We used multivariate Cox proportional hazard regression analysis to compare the risk of glaucoma development between the two groups.

**Results:**

Glaucoma consecutively developed in 4.3% in the CRD group and 2.8% in the control group (*P* < 0.0001). CRD increased the risk of glaucoma development (hazard ratio [HR] = 1.63, 95% confidence interval [CI] = 1.34–1.98] after adjusting for age, sex, comorbidities, residence, household income, and the year of enrollment. In multivariate Cox regression analysis, patients with comorbidity of hypertension, diabetes mellitus, or aged ≥ 50 years showed a significantly higher risk of glaucoma development (all *P* < 0.008).

**Conclusions:**

A significant association between CRD and following development of glaucoma was revealed after adjusting the potential confounding factors.

Glaucoma is an important public-health problem and the second most prevailing cause of irreversible visual impairment worldwide.^1^ Glaucoma is characterized by a progressive injury and remodeling of the optic nerve and a defect of the retinal-nerve fiber layer (RNFL) accompanied by corresponding visual field defects.^2^ Glaucomatous damage leads to the death of retinal ganglion cells (RGCs) and their axons. Glaucoma is recognized to be a multifactorial optic neuropathy and remains a disease of etiology yet unrevealed. Increased intraocular pressure (IOP) is the major risk factor for glaucoma; however, risk factors other than IOP, such as impaired microvascular circulation, vascular injury, and oxidative stress or hypoxia, are also related to the pathogenesis of glaucoma.^3^^–^^5^ Hypoxic situations, which may take place in various ocular disorders, for example, glaucoma, can induce or aggravate apoptosis of RGCs.^6^

Although the pathogenesis of glaucoma is yet unknown, many previous studies reported the association between chronic renal disease (CRD) and glaucoma.^7^^–^^13^ CRD is a well-recognized microvascular disorder involved in renal and cardiovascular implications, which are important public-health issues worldwide. Similar to glaucoma, the prevalence of CRD is increasing rapidly.^14^^,^^15^ Notably, glaucoma and CRD are both closely related to age and to metabolic and cardiovascular risk factors, for example, diabetes and hypertension.^8^^,^^16^^–^^19^ Some previous studies investigated the prevalence of glaucoma in patients with CRD; other studies, vice versa, inspected the prevalence of CRD in patients with glaucoma. Recently, the association between primary open-angle glaucoma (POAG) and following occurrence of CRD was studied with a nationwide, population-based, retrospective cohort in South Korea.^20^ However, there was no previous study evaluating the following risk of glaucoma development in patients with CRD using a longitudinal million-sample cohort of the National Health Insurance Service dataset in South Korea. Considering the serious public-health problem of CRD worldwide and common shared interest with the general physicians, it may be more important to investigate the following incidence of glaucoma in patients with CRD than vice versa. Patients with CRD already have serious systemic impairments, and additional vision-threatening disease like glaucoma could worsen the patients’ quality of life. In the present study, we investigated the risk of subsequent glaucoma development after CRD diagnosis using a representative sample of one million nationwide subjects in a single ethnic group of Asians (Koreans), using the National Health Insurance Service - National Sample Cohort from 2002 to 2013 dataset in South Korea.

## Methods

### Ethics Statement

This study adhered to the tenets of the Declaration of Helsinki, and the NHIS-NCS 2002–2013 project was approved by the Institutional Review Board (IRB) of the Korean National Health Insurance Service. This study was approved by the Institutional Review Board of Gyeongsang National University Changwon Hospital and School of Medicine. An exemption from informed consent for research was granted from the IRB because the study was retrospective.

### Data Sources

In this retrospective cohort study, we used a random sample of 1,025,340 subjects from the National Health Insurance Service - National Sample Cohort 2002 to 2013 dataset, which accounts for approximately 2.2% of the whole population of Korea in the National Health Insurance Service of 2002. We extracted data from the National Health Insurance Service using systematic random sampling for research purposes. Korea has retained a nationwide health insurance system since 1963 under the Korean National Health Insurance Service, and almost all of the data in the health insurance system are integrated in a large data source. Health insurance claims data include diagnostic and procedure codes, prescription of medicines, personal information, and medical care costs. Moreover, the Korean National Health Insurance Service uses the Korean Classification of Diseases (KCD), which resembles the International Classification of Diseases (ICD) in the United States. Data resource profile of the National Health Information Database of the National Health Insurance Service in South Korea has been previously described in detail.^21^

### Study Population

Criteria for inclusion were:

(1) Patients in the study cohort who received medical care between January 1, 2003, and December 31, 2007 with a newly diagnosed CRD (ICD-10, N18, chronic kidney disease, CRD case cohort group); (2) subjects (comparison control cohort) drawn by using 1:5 propensity-score matching from the dataset of 1 million participants without CRD between January 2003 and December 2007; (3) patients newly diagnosed as having glaucoma (ICD-10 code, H40.1, H40.2, H40.3, H40.4, H40.5, and H40.6, glaucoma) who received eyedrop medication (prescribed at ophthalmology) after the diagnosis of CRD from 2003 to 2013 to calculate the main outcome, glaucoma occurrence.

Criteria for exclusion were (1) patients with pre-existing CRD before 2003 and (2) patients with glaucoma occurring before the development of CRD, depending on the date of the visit.

Matching was done with the following variables:

(1) age (< 50/50–59/60–69/70–79/≥ 80 years), (2) sex, (3) household income (≤ 30%, 30–70%, and ≥ 70%), (4) year of enrollment (2003–2007), and (5) residential area.

**Figure 1.**
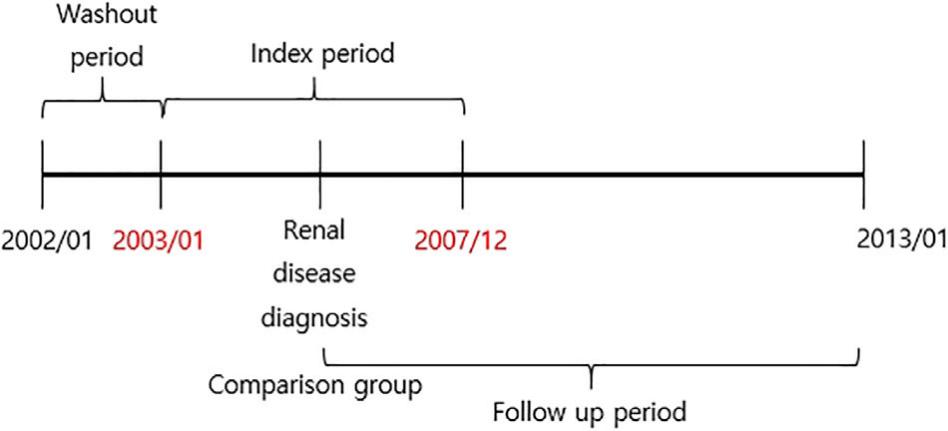
**Study design of the nationwide cohort of one million for 12 years.** There was a 1-year washout period between January 1, 2002 and December 31, 2002. The index period was between January 1, 2003 and December 31, 2007, and newly diagnosed CRD (KCD code N18) was defined as the CRD cohort group. Subjects (comparison control cohort) were extracted using 1:5 propensity-score matching from the database of 1 million subjects without CRD between January 2003 and December 2007. Patients newly diagnosed as having glaucoma (ICD-10 code H40., glaucoma) and who received eyedrop medication (prescribed at ophthalmology) after the diagnosis of CRD were followed from 2003 until 2013 (follow-up period) to assess the outcome variable, glaucoma occurrence.

We did inclusion and exclusion of subjects and propensity matching according to the previously reported methods.^22^^–^^25^ A flowchart about the selection of study subjects is shown in Figure 1.

### Comorbidities

We defined hypertension on the basis of KCD code I10 (corresponding to ICD-9-CM code 401, essential hypertension), diabetes mellitus with KCD code E10 to E14 (corresponding to ICD-9-CM code 250, diabetes mellitus), hyperlipidemia with KCD code E78.0 to E78.5 (corresponding to ICD-9-CM code 272.0, pure hypercholesterolemia; 272.1, pure hyperglyceridemia; 272.2, mixed hyperlipidemia; 272.3, hyperchylomicronemia; and 272.4, other and unspecified hyperlipidemia), and stroke with KCD code I60 to I63 (corresponding to ICD-9-CM code 430–434, cerebrovascular disease) as comorbidities. These comorbidities had to be diagnosed between 2003 and 2013 and prior to the CRD diagnosis in the CRD case-cohort group of matched pairs.

### Statistical Analysis

We carried out propensity-score matching to estimate the occurrence of CRD. We calculated propensity scores using logistic regression to control the socio-demographic factors, including gender, age, household income, and residential area. Residential area was categorized into four areas, including the metropolitan area of Seoul. The second area included the largest province, the third area included the second-largest city and the second and third largest provinces. The fourth area included other areas that was not included in the second and third areas. We carried out matching using the Greedy 8 → 1 digit-matching macro along with the calculated propensity score of each year from 2003 to 2007. In order to calculate the hazards related to CRD, we analyzed hazard ratios (HRs) and 95% confidence intervals (CIs) with univariate and multivariate Cox proportional hazard regression methods. In the multivariate analysis, we performed four modeling methods for the adjusted risk of glaucoma development in CRD. In model 1, only two confounding factors (age groups and sex) were selected. In model 2, the confounding factors which were significant in univariate analysis were selected. In model 3, the confounding factors were selected by the best subset selection method, which is a method that finds the lowest Akaike information criterion (AIC) value among all possible combinations of independent variables. In model 4, was the model that included all independent variables.

We used the Kaplan-Meier curve for the cumulative incidence of glaucoma for each year until 11 years of follow-up periods (2003–2013). If a patient died during the study period, did not develop glaucoma until the end of the study, or did not return to the hospital until the end of the study, the patient was regarded as censored. Follow-up started from the first date of CRD diagnosis or, for the comparison control group, at a randomly selected index date in the matched year. Follow-up finished at the last visit date up to 2013 for subjects without subsequent glaucoma or the first date of glaucoma diagnosis (see Fig. 1). Patients who were newly diagnosed with CRD or glaucoma were only included in the study as described in previous studies.^22^^–^^25^

To test the differences of proportions that are glaucoma events, demographic factors, and other variables, between the control and CRD groups, we performed the Pearson's χ^2^ test. Statistical significance was set at *P* < 0.05. We used the SAS System, version 9.4 (SAS Institute, Inc., Cary, NC, USA) and Stata/MP version 14.0 (StataCorp, College Station, TX, USA) for the statistical analyses.

## Results

### Baseline Characteristics

In total, 21,611 subjects, including 17,971 controls without CRD and 3640 patients with CRD, were eligible for the final analysis. Throughout the whole follow-up period (median = 8.1 years in the control group and median = 6.9 years in the CRD group), glaucoma developed more frequently in subjects with CRD than in those without CRD (4.3% vs. 2.8%, *P* < 0.0001). Comorbidities, such as hypertension, diabetes mellitus, hyperlipidemia, and stroke, were more common in the CRD group than in the socio-demographic-matched control group (*P* < 0.0001, all). There was no significant difference in the proportion of patients according to the presence of CRD for the socio-demographic variables that were used for matching between the two groups (Table 1).

**Table 1.** Characteristics of the Study Population

Variables	Control (*N* = 17,971)	Chronic Renal Disease (N = 3640)	*P* Value
**Glaucoma**			<0.001
**No event**	17,705 (97.3)	3483 (95.7)	
**Event**	495 (2.8)	157 (4.3)	
**Hypertension**			<0.001
**No**	13,203 (72.5)	1334 (36.7)	
**Yes**	4997 (27.5)	2306 (63.4)	
**Diabetes mellitus**			<0.001
**No**	15,053 (82.7)	1921 (52.8)	
**Yes**	3147 (17.3)	1719 (47.2)	
**Hyperlipidemia**			<0.001
**No**	15,574 (85.6)	2211 (60.7)	
**Yes**	2626 (14.4)	1429 (39.3)	
**Stroke**			<0.001
**No**	17,336 (95.3)	3101 (85.2)	
**Yes**	864 (4.7)	539 (14.8)	
**Variables for matching**			
**Year**			0.781
**2003**	3576 (82.8)	745 (17.2)	
**2004**	3559 (82.9)	736 (17.1)	
**2005**	3837 (83.7)	747 (16.3)	
**2006**	3383 (83.2)	682 (16.8)	
**2007**	3616 (83.2)	730 (16.8)	
**Age group (year)**			0.915
**<50**	5085 (83.3)	1017 (16.7)	
**50–59**	3230 (83.3)	646 (16.7)	
**60–69**	4305 (83.3)	861 (16.7)	
**70–79**	3870 (83.3)	774 (16.7)	
**≥80**	1710 (83.3)	342 (16.7)	
**Sex**			0.961
**Male**	9855 (83.3)	1971 (16.7)	
**Female**	8345 (83.3)	1669 (16.7)	
**Residence**			0.999
**Seoul (metropolitan)**	4200 (83.3)	840 (16.7)	
**Second area**	1425 (83.3)	285 (16.7)	
**Third area**	1820 (83.3)	364 (16.7)	
**Fourth area**	10,755 (83.3)	2151 (16.7)	
**Household income**			0.995
**0–30%**	4170 (83.3)	834 (16.7)	
**30–70%**	6185 (83.3)	1237 (16.7)	
**70–100%**	7845 (83.3)	1569 (16.7)	

*P* value by Pearson's χ^2^ test.

Percent proportion is indicated in parentheses.

### Factors Associated With Glaucoma Incidence

Univariate Cox proportional hazard regression results are shown in Table 2. We built four multivariate models, including the one with all variables adjusted (model 4). We found that glaucoma was associated with a following diagnosis of CRD (HR = 1.63, 95% CI = 1.34–1.98) in model 4 and in all the other 3 models (HR = 2.1 in model 1, HR = 1.63 in model 2, and HR = 1.61 in model 3). Comorbidity of hypertension (HR = 1.48, 95% CI = 1.23–1.78) and diabetes mellitus (HR = 1.52, 95% CI = 1.26–1.83) were significantly associated with glaucoma development in our multivariate model 4 and also significant in model 2 and in model 3, (all *P* < 0.001; Table 3). However, other comorbidities, such as hyperlipidemia and stroke, were not significantly associated with glaucoma development in model 4 and also not in model 2 (all *P* > 0.05).

**Table 2.** Univariable Cox Hazard Proportion Analysis for the Overall Incidence of Glaucoma

	Glaucoma		Univariable Cox	
Variables	No Event (*N* = 20,959)	Event (*N* = 652)	HR (95% CI)	*P* Value
**Group**				
**Control**	17,476 (97.3)	495 (2.8)	1.00	
**Chronic renal disease**	3483 (95.7)	157 (4.3)	1.98 (1.66–2.37)	<0.001
**Hypertension**				
**No**	14,086 (97.8)	322 (2.2)	1.00	
**Yes**	6873 (95.4)	330 (4.6)	2.56 (2.19–2.99)	<0.001
**Diabetes mellitus**				
**No**	16,407 (97.6)	412 (2.5)	1.00	
**Yes**	4552 (95.0)	240 (5.0)	2.55 (2.18–3.00)	<0.001
**Hyperlipidemia**				
**No**	17,117 (97.3)	473 (2.7)	1.00	
**Yes**	3842 (95.6)	179 (4.5)	1.93 (1.62–2.29)	<0.001
**Stroke**				
**No**	19,640 (97.0)	604 (3.0)	1.00	
**Yes**	1319 (96.5)	48 (3.5)	1.65 (1.23–2.22)	0.001
**Age group (year)**				
**<50**	6023 (98.7)	77 (1.3)	1.00	
**50–59**	3752 (97.1)	113 (2.9)	2.43 (1.82–3.25)	<0.001
**60–69**	4911 (95.6)	227 (4.4)	3.91 (3.02–5.06)	<0.001
**70–79**	4356 (95.5)	204 (4.5)	4.60 (3.53–5.98)	<0.001
**≥80**	1917 (98.4)	31 (1.6)	2.24 (1.48–3.41)	0.001
**Sex**				
**Male**	11,373 (97.3)	321 (2.7)	1.00	
**Female**	9586 (96.7)	331 (3.3)	1.16 (0.99–1.35)	0.062
**Residence**				
**Seoul (metropolitan)**	4825 (96.6)	169 (3.4)	1.00	
**Second area**	1644 (97.3)	46 (2.7)	0.83 (0.6–1.16)	0.275
**Third area**	2121 (97.9)	45 (2.1)	0.60 (0.43–0.83)	0.002
**Fourth area**	12,369 (96.9)	392 (3.1)	0.92 (0.77–1.1)	0.375
**Household income**				
**0–30%**	4818 (97.3)	135 (2.7)	1.00	
**30–70%**	7141 (97.1)	217 (2.9)	1.04 (0.84–1.29)	0.702
**70–100%**	9000 (96.8)	300 (3.2)	1.18 (0.96–1.44)	0.118

**Table 3.** Multivariable Cox Hazard Proportion Analysis for the Overall Incidence of Glaucoma by Different Modeling

	Model 1		Model 2		Model 3		Model 4	
Variables	Adjusted HR (95% CI)	*P* Value	Adjusted HR (95% CI)	*P* Value	Adjusted HR (95% CI)	*P* Value	Adjusted HR (95% CI)	*P* Value
**Group**								
**Control**	1.00		1.00		1.00		1.00	
**Chronic renal disease**	2.1 (1.76–2.52)	<0.001	1.63 (1.34–1.98)	<0.001	1.61 (1.33–1.96)	<0.001	1.63 (1.34–1.98)	<0.001
**Hypertension**								
**No**			1.00		1.00		1.00	
**Yes**			1.48 (1.23–1.78)	<0.001	1.46 (1.22–1.75)	<0.001	1.48 (1.23–1.78)	<0.001
**Diabetes mellitus**								
**No**			1.00		1.00		1.00	
**Yes**			1.51 (1.25–1.83)	<0.001	1.51 (1.26–1.81)	<0.001	1.52 (1.26–1.83)	<0.001
**Hyperlipidemia**								
**No**			1.00				1.00	
**Yes**			1.01 (0.83–1.24)	0.900			1.00 (0.83–1.23)	0.940
**Stroke**								
**No**			1.00				1.00	
**Yes**			0.82 (0.61–1.12)	0.218			0.83 (0.61–1.13)	0.241
**Age group (year)**								
**<50**	1.00		1.00		1.00		1.00	
**50–59**	2.47 (1.85–3.30)	<0.001	2.09 (1.56–2.80)	<0.001	2.09 (1.56–2.81)	<0.001	2.10 (1.56–2.81)	<0.001
**60–69**	4.01 (3.09–5.19)	<0.001	3.08 (2.35–4.04)	<0.001	3.07 (2.35–4.03)	<0.001	3.11 (2.37–4.07)	<0.001
**70–79**	4.78 (3.68–6.22)	<0.001	3.54 (2.68–4.69)	<0.001	3.49 (2.64–4.61)	<0.001	3.56 (2.69–4.71)	<0.001
**≥80**	2.36 (1.55–3.58)	<0.001	1.80 (1.17–2.76)	0.007	1.75 (1.14–2.69)	0.010	1.79 (1.16–2.75)	0.008
**Sex**								
**Male**	1.00				1.00		1.00	
**Female**	1.16 (0.99–1.35)	0.062			1.16 (0.99–1.35)	0.065	1.15 (0.99–1.35)	0.069
**Residence**								
**Seoul (metropolitan)**			1.00		1.00		1.00	
**Second area**			0.82 (0.59–1.14)	0.230	0.83 (0.60–1.15)	0.263	0.82 (0.59–1.14)	0.247
**Third area**			0.66 (0.48–0.92)	0.015	0.66 (0.48–0.92)	0.015	0.66 (0.48–0.92)	0.015
**Fourth area**			0.93 (0.77–1.11)	0.425	0.93 (0.77–1.11)	0.422	0.93 (0.77–1.11)	0.423
**Household income**								
**0–30%**							1.00	
**30–70%**							1.08 (0.87–1.34)	0.477
**70–100%**							1.03 (0.84–1.26)	0.775
**Model criterion value**	AUC: 0.668; AIC: 12451.1		AUC: 0.696; AIC: 12405.6		AUC: 0.696; AIC: 12401.7		AUC: 0.697; AIC: 12407.7	

AUC, area under the curve; AIC, Akaike's Information Criterion; the model with the largest AUC or lowest AIC value being considered the best.

Model 1 adjusted for age and sex. Model 2 adjusted for confounding factors which were significant in univariate analysis. Model 3 adjusted for the confounding factor which were selected by the best subset selection method. It is a method that finds the lowest Akaike information criterion (AIC) value among all possible combinations of independent variables. Model 4 adjusted for all independent variables.

The age group of more than 50 years old showed significantly higher incidence of glaucoma than did the age group of less than 50 years old as the reference group (all *P* < 0.010). The adjusted HR of age 50 to 59 years was 2.10 (1.56–2.81), age 60 to 69 years was 3.11 (2.37–4.07), age 70 to 79 years was 3.56 (2.69–4.71), and an age of more than 80 years was 1.79 (1.16–2.75) in model 4, and all the other models showed consistently the same tendency (all *P* < 0.05). Men and women did not show significant differences in the incidence of glaucoma in our calculated study subjects in model 4 (*P* = 0.069) and neither in model 1 or in model 3 (see Table 3).

We analyzed residential area, with metropolitan Seoul as the reference group. The second and fourth areas did not show any significant difference in the development of glaucoma in all models (all *P* > 0.230). However, the third area showed significantly less development of glaucoma, with HR of 0.66 in all models (all *P* = 0.015). Household income showed no significant differences among 0% to 30%, 30% to 70%, and 70% to 100% in model 4 (all *P* > 0.477; see Table 3).

### Cumulative Incidence for Glaucoma

**Figure 2.**
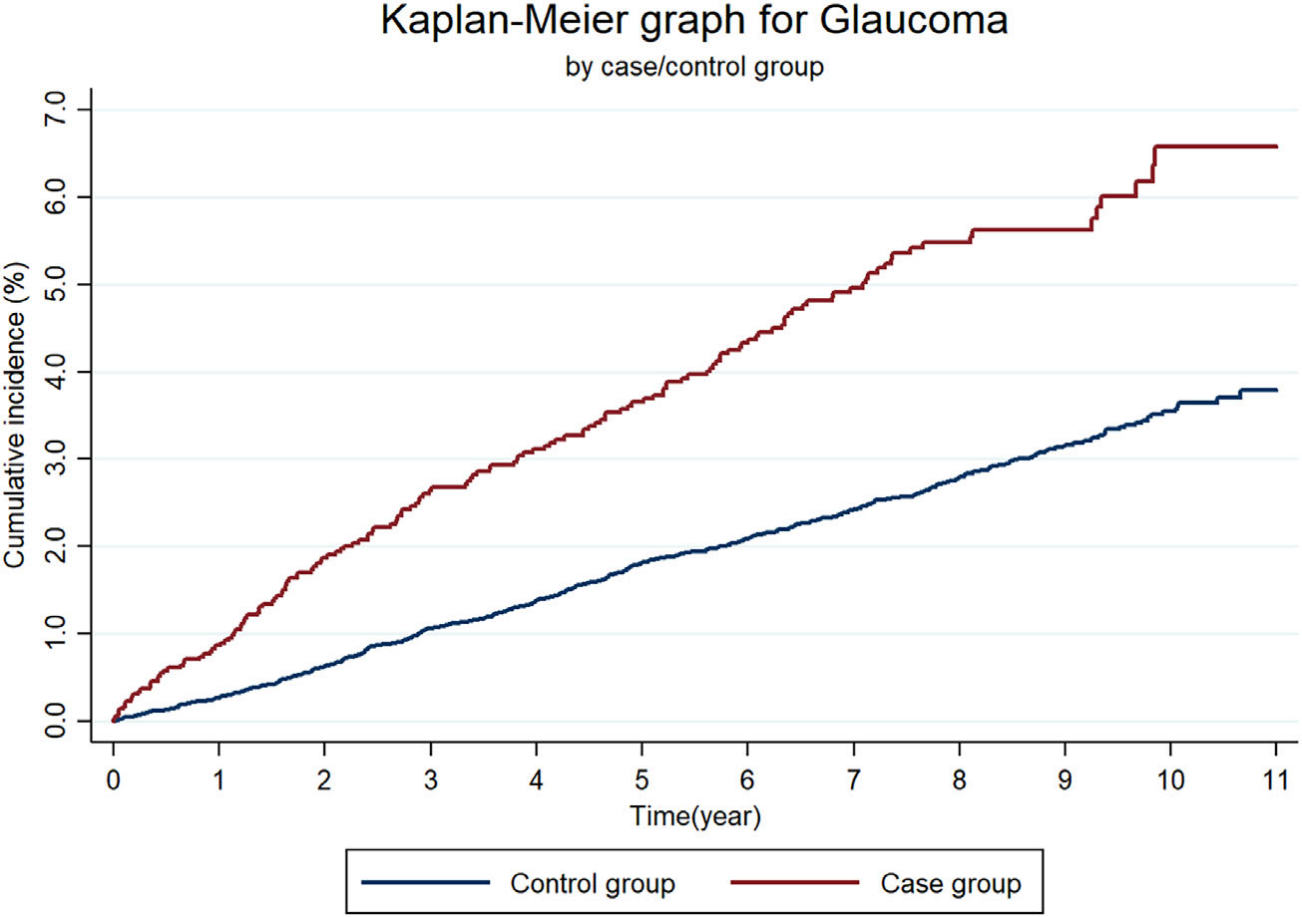
**Cumulative incidence of glaucoma for up to 11 years by Kaplan-Meier survival curves.** Those with chronic renal disease (CRD) had an occurrence of glaucoma more frequently than did the control group without CRD. Kaplan-Meier survival curves for the CRD group and control group showed significant differences using the log-rank test (*P* < 0.0001). Cumulative incidence of glaucoma at 11 years was significantly higher in the CRD group (6.6%) than in the control group without CRD (3.8%). Cumulative incidence of glaucoma in the CRD group was higher than that in the control group at each time point during the follow-up period of 11 years.

Kaplan-Meier curves for the cumulative incidence of glaucoma in each year for up to 11 years are shown in Figure 2. Those with CRD had glaucoma more frequently than did the control group without CRD. Kaplan-Meier curves for the CRD group and control group showed a significant difference using the log-rank test (*P* < 0.0001). Cumulative incidence of glaucoma at 11 years was significantly higher in the CRD group (6.6%) than in the control group without CRD (3.8%). The cumulative incidence of glaucoma at each time point of each year during the 11 years of follow-up is described in the Supplementary Table; in the CRD group and control group at 3 years, the incidence was 2.6% and 1.1%, respectively, at 5 years it was 3.7% and 1.8%, respectively, at 7 years it was 5.0% and 2.4%, respectively, and at 10 years it was 6.6% and 3.5%, respectively. The cumulative incidence of glaucoma in the CRD group was higher than that in the control group at each time point during the follow-up period of 11 years.

## Discussion

To the best of our knowledge, this study is the first to demonstrate the significant association between newly diagnosed CRD and subsequent glaucoma development using a cohort of one million with long-term follow-up of 12 years based on the Korean National Health Insurance Service dataset. Because CRD is rapidly increasing worldwide and glaucoma is the second-most common cause of irreversible visual loss, both CRD and glaucoma are of significant social importance in terms of public health care. We found that subsequent glaucoma developed much more frequently in subjects with newly diagnosed CRD than in those without CRD as the control. The cumulative incidence of glaucoma was significantly higher in subjects with CRD than in the control at 11 years of follow-up.

In a previous population-based study using the Korean National Health and Nutrition Examination Survey (KNHANES) 2010 to 2011, impaired renal function, defined as the estimated glomerular filtration rate (eGFR) lower than 60 mL/min/1.73 m^2^, was independently associated with the prevalence of POAG.^12^ Another previous study also using the KNHANES 2011 to 2012 reported that albuminuria, even low-grade, was significantly associated with OAG in nondiabetic subjects.^26^ These results are concordant with the main findings of our study that CRD increased the risk of following glaucoma development in the Korean population, although our study was a longitudinal study compared with the cross-sectional previous studies. Another population-based study using the Taiwan Longitudinal Health Insurance Database 2000, they found that chronic renal failure (CRF) had a significantly higher prevalence of glaucoma and other eye diseases, including retinal disorders, uveitis, and cataract, compared with patients without CRF.^9^ This study had a similar design of large-scale population-based study, including 1,000,000 beneficiaries of the health insurance database, but the data were analyzed in the cross-sectional manner. Our study provides the longitudinal cumulative incidence of glaucoma for 11 years of follow-up, and, therefore, it has a unique meaning among other previous population-based studies.

Common mechanisms of pathophysiology underlying both CRD and glaucoma are considered to be renin-angiotensin system (RAS) dysfunction, oxidative stress, atherosclerosis, and inflammation. RAS has an important role in the control of blood pressure (BP) and in the homeostasis of electrolytes. Ocular RAS has been observed in aqueous humor, trabecular meshwork, ciliary body, and the optic disc.^27^ It has been reported that ocular RAS may have an essential role in the regulation of IOP through aqueous-humor production and drainage pathways.^27^^–^^29^ Furthermore, an angiotensin-converting enzyme inhibitor (captopril) and an angiotensin II type 1 receptor antagonist (candesartan) demonstrated neuroprotective effects against RGC damage in an animal glaucoma model.^30^ Because ocular RAS affects IOP regulation and RGCs, ocular RAS may be a keystone in the pathogenesis of glaucoma and a potential target for glaucoma treatment as well.^27^^–^^29^

Oxidative stress is also a common mechanism of pathophysiology concerned in both CRD and glaucoma.^5^^,^^6^^,^^31^^,^^32^ The role of oxidative stress on the pathogenesis of glaucoma has been well recognized.^5^^,^^6^^,^^31^ Oxidative stress can lead to the death of RGCs eventually and results in glaucomatous optic neuropathy. Moreover, in CRD, oxidative stress has an essential role in the course of renal fibrosis.^32^ These common mechanisms between CRD and glaucoma may have influenced the following development of glaucoma in patients with CRD, as demonstrated in our study results.

In patients with CRD, osmotic pressure exerted by increased urea concentration in the aqueous humor may result in fluid overload in the anterior chambers of the eyes.^33^ Moreover, toxic metabolites that accumulate in the trabecular meshwork may block aqueous outflow.^34^ The relationship between IOP and glaucoma in patients with CRD were reported to be variable among population-based studies.^8^ In these regards, it has been suggested that other non-IOP factors, such as ischemia and neuroprotective mechanisms, may affect the susceptibility of the optic nerve to pressure-related damage.^8^

Several studies using the national health claims data from different countries demonstrated the association between systemic hypertension and glaucoma.^22^^,^^35^^,^^36^ The study using the National Danish Registry of Medicinal Products Statistics reported that patients treated with antihypertensive medication had a significantly higher overall relative risk (RR) of glaucoma, even when controlling for age and sex (with an RR of 1.31 and *P* < 0.0001).^35^ They additionally showed the causal effect of antihypertensive medication on the delayed onset of glaucoma.^36^ Another study using the Taiwan's nationwide health insurance claims data also revealed that POAG was significantly associated with prior systemic hypertension (odds ratio = 1.31, 95% CI = 1.29–1.33) after adjusting for all confounding factors.^36^ Using the same data from the Korean National Health Insurance Service, Rim et al. showed that patients with hypertension were more prone to have following OAG than are those without hypertension.^22^ The proportion of patients with hypertension was significantly higher in the CRD group than in the control group without CRD in our CRD case-control study (*P* < 0.0001). Moreover, in our multivariate Cox regression model, comorbidity of systemic hypertension was also significantly associated with development of glaucoma (HR = 1.48, *P* < 0.0001). One possible mechanism of hypertension involved in the development of glaucoma is microvascular circulatory disturbances related to the decrease of ocular perfusion to the optic disc.^37^ Because hypertension is a strong risk factor for CRD due to the microvasculopathy caused by hypertension,^8^^,^^16^^,^^18^ it seems reasonable that the comorbidity of hypertension in CRD showed a more significant development of glaucoma than in those without hypertension in the present study.

It has been previously reported that diabetes and type 2 diabetes mellitus were associated with subsequent glaucoma development, using the same Korean National Health Insurance Service data.^23^^,^^24^ Patients with diabetes are affected by several factors, such as oxidative stress,^38^ advanced glycation end products,^39^ and obstructed retrograde axonal flow of RGCs.^40^ In this study, comorbidity of diabetes was significantly related to development of glaucoma (HR = 1.52, *P* < 0.0001). Diabetes mellitus is a well-known disorder engaged in microvascular implications, because it induces structural and functional injury to small blood vessels, which can impair microvascular circulation of the optic nerve and retina.^41^ These mechanisms may have influenced the results of our study regarding comorbidity of diabetes.

Patients with CRD already have serious systemic impairments, and some patients are even on hemodialysis. Moreover, many patients with CRD have comorbidity of diabetes or hypertension and, thus, already have a high risk of diabetic or hypertensive retinopathy, which could lead to visual loss. Additional vision-threatening disease like glaucoma could worsen the patients’ quality of life, for example, contributing to motor disturbances like falling, which could lead to another medical treatment. Therefore, it may be important for nephrologist to consider referring these patients with CRD to ophthalmologists, especially to a glaucoma specialist for proper evaluation and management to prevent further visual impairment and medical complications.

The strength of this study is that it included one million national cohort subjects with a long-term follow-up of 12 years. We are not aware of any previous study that investigated glaucoma development following CRD using Korean National Health Insurance Service data.

There are several limitations of this study, the most important being the possible incorrectness of the diagnoses of CRD and glaucoma based on KCD codes. However, there are several published papers using Korean National Health Insurance claims data for glaucoma.^20^^,^^22^^–^^24^ Furthermore, the cumulative incidence of glaucoma in the control group consistently increased for 11 years in Figure 2; this may partly indicate the validity of the glaucoma diagnosis in this study. Second, glaucoma may have been underdiagnosed and under-reported, because it may be asymptomatic until a relatively late stage; hence diagnosis of glaucoma is often delayed or missed at an early stage because of delayed visits to ophthalmologists. These cases may have belonged to the non-glaucoma event group; therefore, the real HR may be greater than the HR presented in this study. Third, glaucoma diagnoses were not subclassified according to subtypes. The KCD code-based diagnosis does not always reflect the correct cause of glaucoma, because gonioscopy or anterior segment optical coherence tomography was not consistently used to distinguish angle status. IOP was also not always measured with Goldmann applanation tonometry, and it is difficult to discriminate normal-tension glaucoma with only the KCD code. In this regard, we included overall glaucoma with KCD code-based diagnoses. However, the mechanisms of the included glaucoma may be different according to subtypes of glaucoma.

As other limitations of our study, the following should be mentioned:

(1) Other data of health examinations, such as the body mass index and behavioral risk factors, such as smoking or alcohol status, were only partially included. In this aspect, these potential confounding factors could not be adjusted.
(2) There was a higher probability of bias between the control group patients from the health insurance data and healthy control patients from the general population who were not registered for health insurance data without having received any medical care.
(3) Potential discrepancies may exist in ethnic groups other than this Korean population, a possibility not regarded in the present study.

In conclusion, CRD was significantly associated with subsequent glaucoma development after adjusting for potential confounding factors. Physicians should pay careful attention to patients with CRD, especially those with comorbidities of hypertension and diabetes, and consider referring the patient to an ophthalmologist for glaucoma screening to avoid potential impairment of vision. We should be aware that additional vision-threatening disease like glaucoma could worsen the quality of life of these patients with CRD, who already have systemic impairments. Population-based multicenter studies are required for a definitive conclusion.

## Supplementary Material

Supplement 1
