# Supplementary material for: Association Between Chronic Renal Disease and the Risk of Glaucoma Development: A 12-year Nationwide Cohort Study
Source: Invest Ophthalmol Vis Sci. 2021 May 27;62(6):27. doi: 10.1167/iovs.62.6.27 (PMC8164364; doi:10.1167/iovs.62.6.27)
Supplement: Supplement 1 [file iovs-62-6-27_s001.pdf]

SUPPLEMENTARY TABLE. Cumulative Incidence (%) of Glaucoma during the Follow-Up Period of 11 Years

	N	Event	Cumulative incidence (%) at 1 year	Cumulative incidence (%) at 2 years	Cumulative incidence (%) at 3 years	Cumulative incidence (%) at 4 years	Cumulative incidence (%) at 5 years	Cumulative incidence (%) at 6 years	Cumulative incidence (%) at 7 years	Cumulative incidence (%) at 8 years	Cumulative incidence (%) at 9 years	Cumulative incidence (%) at 10 years	Cumulative incidence (%) at 11 years
**Total**	21,611	652	0.36 (0.28 - 0.44)	0.83 (0.70 - 0.95)	1.30 (1.15 - 1.46)	1.64 (1.47 - 1.82)	2.10 (1.90 - 2.30)	2.42 (2.21 - 2.64)	2.79 (2.56 - 3.02)	3.19 (2.93 - 3.44)	3.52 (3.24 - 3.81)	3.97 (3.64 - 4.31)	4.19 (3.80 - 4.58)
**Group**													
**Control**	17,971	495	0.27 (0.19 - 0.35)	0.64 (0.52 - 0.75)	1.06 (0.91 - 1.21)	1.38 (1.21 - 1.56)	1.83 (1.63 - 2.03)	2.09 (1.88 - 2.31)	2.42 (2.19 - 2.66)	2.80 (2.54 - 3.06)	3.16 (2.87 - 3.46)	3.54 (3.20 - 3.89)	3.79 (3.38 - 4.20)
**Chronic Renal disease**	3,640	157	0.86 (0.55 - 1.17)	1.87 (1.40 - 2.34)	2.64 (2.07 - 3.20)	3.11 (2.50 - 3.73)	3.65 (2.97 - 4.33)	4.33 (3.59 - 5.08)	4.97 (4.16 - 5.78)	5.49 (4.61 - 6.36)	5.63 (4.73 - 6.53)	6.58 (5.41 - 7.76)	6.58 (5.41 - 7.76)

Kaplan-Meier analysis

95% confidence interval is indicated in ( ).
